# Integrating drone-borne thermal imaging with artificial intelligence to locate bird nests on agricultural land

**DOI:** 10.1038/s41598-020-67898-3

**Published:** 2020-07-14

**Authors:** Andrea Santangeli, Yuxuan Chen, Edward Kluen, Raviteja Chirumamilla, Juha Tiainen, John Loehr

**Affiliations:** 1 The Helsinki Lab of Ornithology, Finnish Museum of Natural History, University of Helsinki, 00014 Helsinki, Finland; 2 FitzPatrick Institute of African Ornithology, DST-NRF Centre of Excellence, University of Cape Town, Cape Town, South Africa; 3 Helsinki Institute of Sustainability Science, University of Helsinki, 00014 Helsinki, Finland; 4 Lammi Biological Station, Faculty of Biological and Environmental Sciences, University of Helsinki, Pääjärventie 320, 16900 Lammi, Finland; 5 Department of Electrical and Electronic Engineering, Imperial College London, South Kensington Campus, Greater London, SW7 2AZ UK; 6 HiLIFE Helsinki Institute of Life Science, University of Helsinki, 00014 Helsinki, Finland; 7 Research Program in Organismal and Evolutionary Biology, Faculty of Biological and Environmental Sciences, University of Helsinki, Helsinki, Finland; 8 Sir C R Reddy College of Engineering, Andhra University, Eluru, Andhra Pradesh 534007 India; 9 Natural Resources Institute Finland (Luke), PL 2, 00791 Helsinki, Finland

**Keywords:** Ecology, Zoology, Ecology, Environmental sciences

## Abstract

In conservation, the use of unmanned aerial vehicles (drones) carrying various sensors and the use of deep learning are increasing, but they are typically used independently of each other. Untapping their large potential requires integrating these tools. We combine drone-borne thermal imaging with artificial intelligence to locate ground-nests of birds on agricultural land. We show, for the first time, that this semi-automated system can identify nests with a high performance. However, local weather, type of arable field and height of the drone can affect performance. The results’ implications are particularly relevant to conservation practitioners working across sectors, such as biodiversity conservation and food production in farmland. Under a rapidly changing world, studies like this can help uncover the potential of technology for conservation and embrace cross-sectoral transformations from the onset; for example, by integrating nest detection within the precision agriculture system that heavily relies on drone-borne sensors.

## Introduction

Biodiversity is being lost at unprecedented rates, while pervasive lack of knowledge, such as information on species’ ecology, life-history and distribution hamper conservation^1,2^. At the same time, available resources allow very limited monitoring of biodiversity. This is partly due to the challenges in detecting and identifying species using traditional methods, e.g. direct observations. Traditional survey methods are typically laborious, requiring large workforce, time and resources, and may be affected by an observer bias^3,4^. Therefore, there is an urgent need for efficient biodiversity surveys that can inform conservation.

As advanced technologies become more affordable, they are increasingly used by ecologists and conservation practitioners to monitor species and ecosystems^5^. Given the massive environmental challenges the world is facing, it is paramount that the power of technology to aid conservation is fully unleashed through innovation, transdisciplinary and transboundary cooperation^6^.

Remote sensing technology, such as unmanned aircraft systems (hereafter drones) are becoming popular in the environmental community as effective and cost-saving options for field-based work, such as detecting and monitoring wildlife and their habitats (e.g.^3,4,7–9^). While widely perceived as a promising tool for surveying wildlife, drone technology combined with various associated sensors, such as thermal or RGB cameras, has been applied in a very narrow range of conditions. Such cases include surveying large animals^3,4,10^ or locating birds’ nests^9,11,12^. However, most such attempts have interpreted the large number of resulting images manually (e.g.^12^).

Technology allows collecting large amounts of data. Thus, to fully exploit the potential of using technology in conservation and make it more efficient than traditional approaches, it is crucial that the resulting information is processed in a semi- or fully automated way. Doing so would allow the scaling up of technological innovations to monitor wildlife and provide the information necessary for practical management. Recent research has made steps in the above direction. For example, thermal images acquired from a drone have been used to identify bird nests in grassland ecosystems^12^, however, manual identification of nests in thermal images limited the efficiency of this method. Automation of this type of fieldwork was demonstrated by^3^ where they combined drone-borne aerial surveys using a thermal camera with machine learning to identify an elusive and difficult to survey large arboreal mammal, the koala (*Phascolarctus cinereus*).

Drones are being currently used in many different fields, including agriculture. Specifically, the recent move towards precision agriculture heavily relies on drone technology paired with various sensors for in-depth field monitoring and mapping^13^. Providing a scalable technological solution that integrates automated location of ground nests of birds into routine field monitoring would ultimately aid nest protection and wildlife monitoring. This would help to reduce the overall impacts of agriculture on wildlife.

Here we test for the first time, the effectiveness of a semi-automated system based on a thermal sensor carried by a drone and a deep learning algorithm to efficiently and accurately detect ground nests of farmland birds while assessing the impact of local weather, environmental conditions and drone height on detection accuracy. We use nests of a farmland bird species, the northern lapwing *Vanellus vanellus* (hereafter lapwing), a ground nesting species whose nests, as well as those of other similar farmland birds, are often destroyed by mechanical farming operations. Such nests are also very challenging and laborious to locate, hindering their protection. A semi-automated nest identification system could thus greatly boost the conservation of these farmland bird species, which are collectively and steeply declining across most of Europe and beyond^14^.

## Results

**Figure 1:**
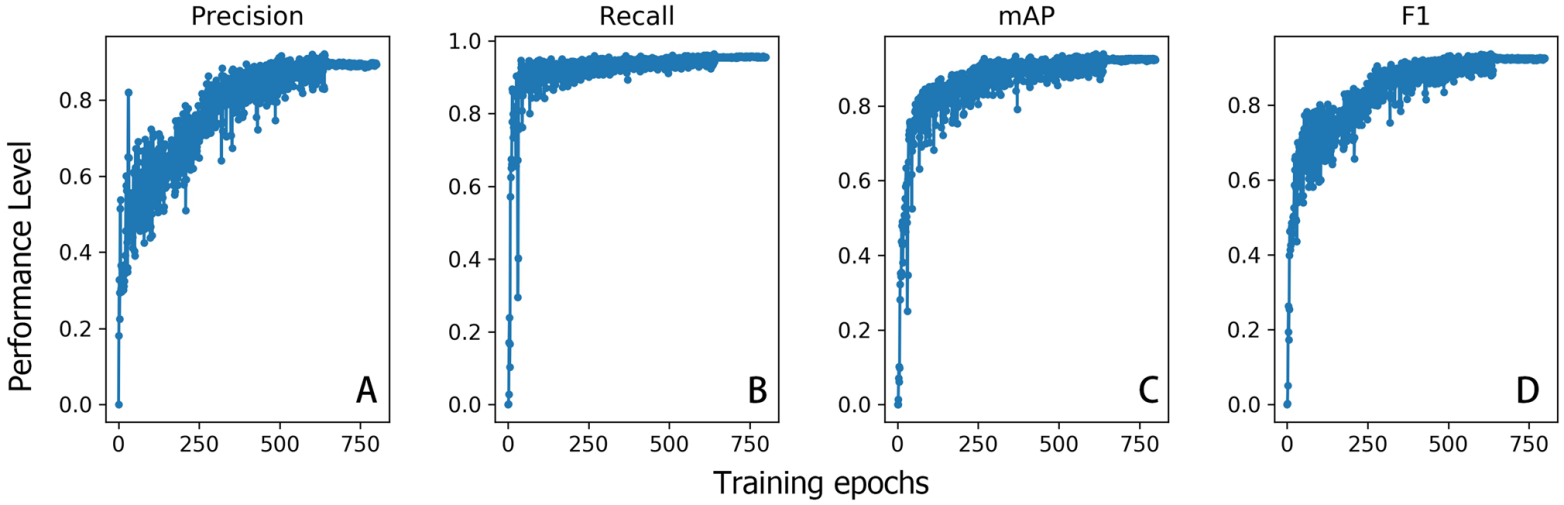
Training performance as measured by the proportion of images correctly identified by the neural network in relation to the number of epochs. Performance is shown based on four metrics: Precision (**A**), recall (**B**), mAP (**C**) and F1 score (**D**). See “Methods” section for a detailed description of each of the performance metrics. Figure created in R software version 3.6.1 (www.r-project.org).

The training performance of the neural network started to converge towards high levels after about 600 epochs, reaching 89.4% precision, 95.6% recall, 92.4% mAP and 92.4% F1 at the final epoch on training images (Fig. 1). Evaluation of the neural network system on the “unseen” testing images indicated high precision levels (97.8%), i.e. the system is very good at avoiding false positives, but a less high recall (82.7%), i.e. avoiding false absences (see also Figure S2 and S3).

**Figure 2:**
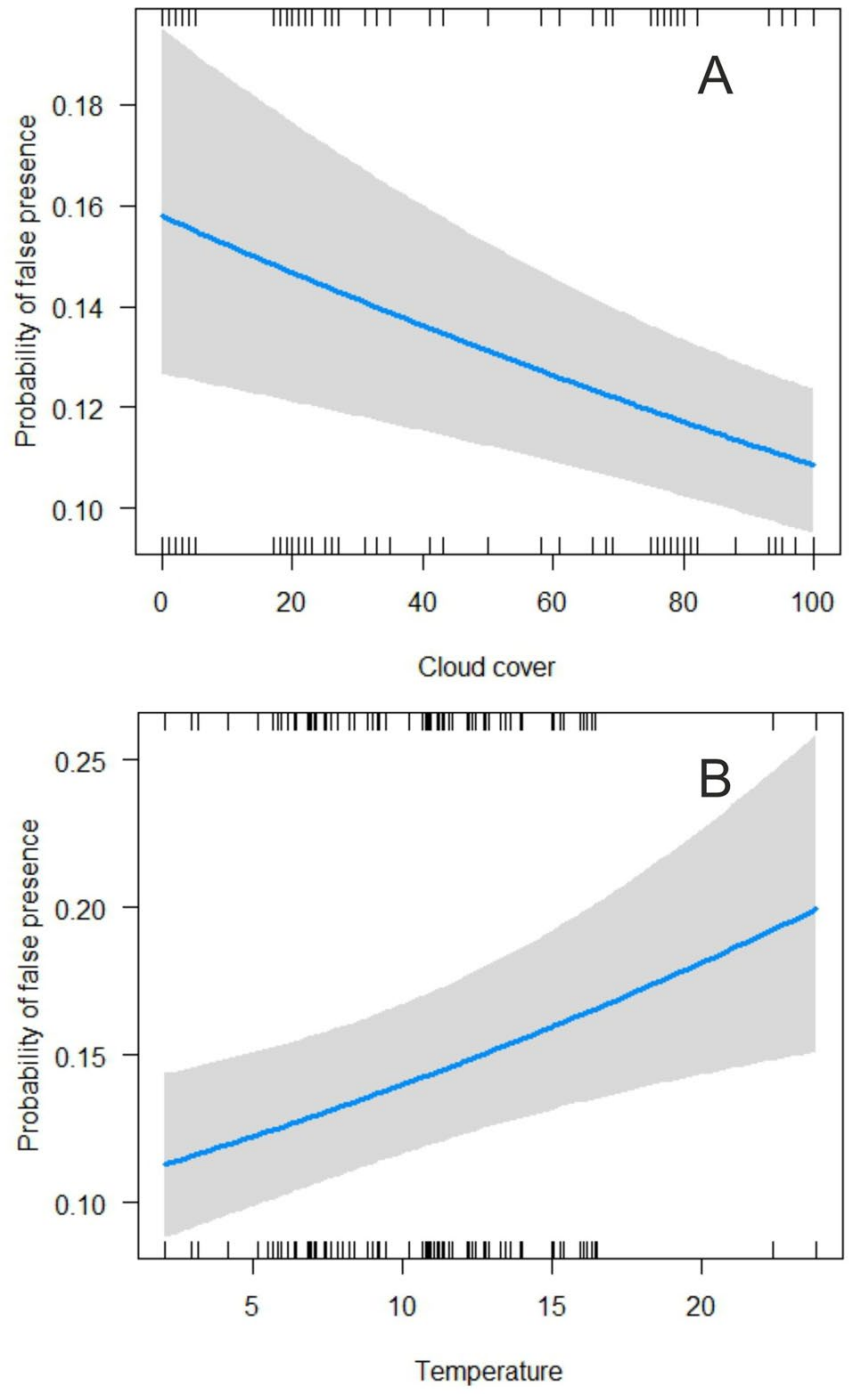
The probability of occurrence of false presences, whereby the deep learning algorithm erroneously identified a nest in images without a nest, in relation to cloud cover (**A**), and air temperature (**B**). Lines depict mean probability and associated 95% confidence interval (grey shading) as derived from the best supported model obtained from model selection (results shown in Table 1). Data are shown by the ticks on the horizontal axes. Figure created in R software version 3.6.1 (www.r-project.org).

The factors that most affect occurrence of false presences are temperature, cloud cover and substrate type (Table 1). Specifically, probability of false presence is higher in conditions of high temperature, low cloud cover (Fig. 2), and on ploughed compared to un-ploughed fields (least square means: 0.16 ± 0.01 SE and 0.14 ± 0.01 SE, respectively). Probability of false absences was only affected by the height of the drone, being two times higher when images were taken from 25 m above ground level compared to 15 m (least square means: 0.38 ± 0.11 SE and 0.18 ± 0.03 SE, respectively).

**Table 1:** The relationship between probability of occurrence of false presences (A) and false absences (B) and a set of uncorrelated environmental covariates.

	β	SE	z	p
**(A) False presence**				
Intercept	− 1.767	0.133	13.26	< 0.001
Cloud cover	− 0.005	0.001	3.63	< 0.001
Temperature	0.028	0.011	2.50	0.013
Wind speed	0.020	0.018	1.09	0.276
Substrate (un-ploughed)	− 0.199	0.089	2.24	0.025
Drone height (25 m)	− 0.053	0.087	0.61	0.542
**(B) False absence**				
Intercept	− 0.867	0.737	1.18	0.240
Cloud cover	− 0.002	0.007	0.31	0.759
Temperature	− 0.098	0.062	1.58	0.113
Substrate (un-ploughed)	− 0.453	0.392	1.15	0.250
Drone height (25 m)	0.974	0.460	2.12	0.035

Effect size (β), standard error (SE), test statistics (z) as well as *p* values are derived from averaging across the set of best supported models (reported in Table S1) run separately for false presence and false absence (see “Methods” section for more details). For the two categorical variables with two classes each, results consider un-ploughed fields as a reference for the substrate variable (compared to ploughed substrate), and height of 25 m for the drone height variable (compared to 15 m height).

## Discussion

For the first time we show that a semi-automated system based on drone-borne thermal imaging and deep learning can accurately identify nests of a medium-sized ground-nesting bird on arable land. The evaluation metrics indicate a high performance of the system, which has a high capability in classifying the thermal images and identifying those with a lapwing nest. However, we show that local weather, type of arable field and height of the drone can affect performance.

Overall, the performance (precision and recall on both learning and testing images) reported here is among the highest for studies using automated methods to detect wildlife (see^3^ and references therein). This high performance is likely due to the open and structurally simple environment, and to the fact that the target object, the lapwing nest, is consistent in shape and size. However, conditions of high cloud cover, low temperature and un-ploughed fields of even surface significantly minimize the occurrence of false presences. These results are likely due to the fact that high irradiation and temperature warm objects resulting in them developing a similar thermal signature as a lapwing nest. Similarly, ploughed fields are irregular in structure and may contain exposed objects, such as stones, that may thermally resemble lapwing nests. The above findings, coupled with the significant reduction in false absences when the image was taken from lower altitude, underscore difficult trade-offs when implementing the system on the ground. An optimal balance between minimizing false presences and false absences should be sought in each case by defining the threshold confidence level for detection based on expected outcomes.

**Figure 3:**
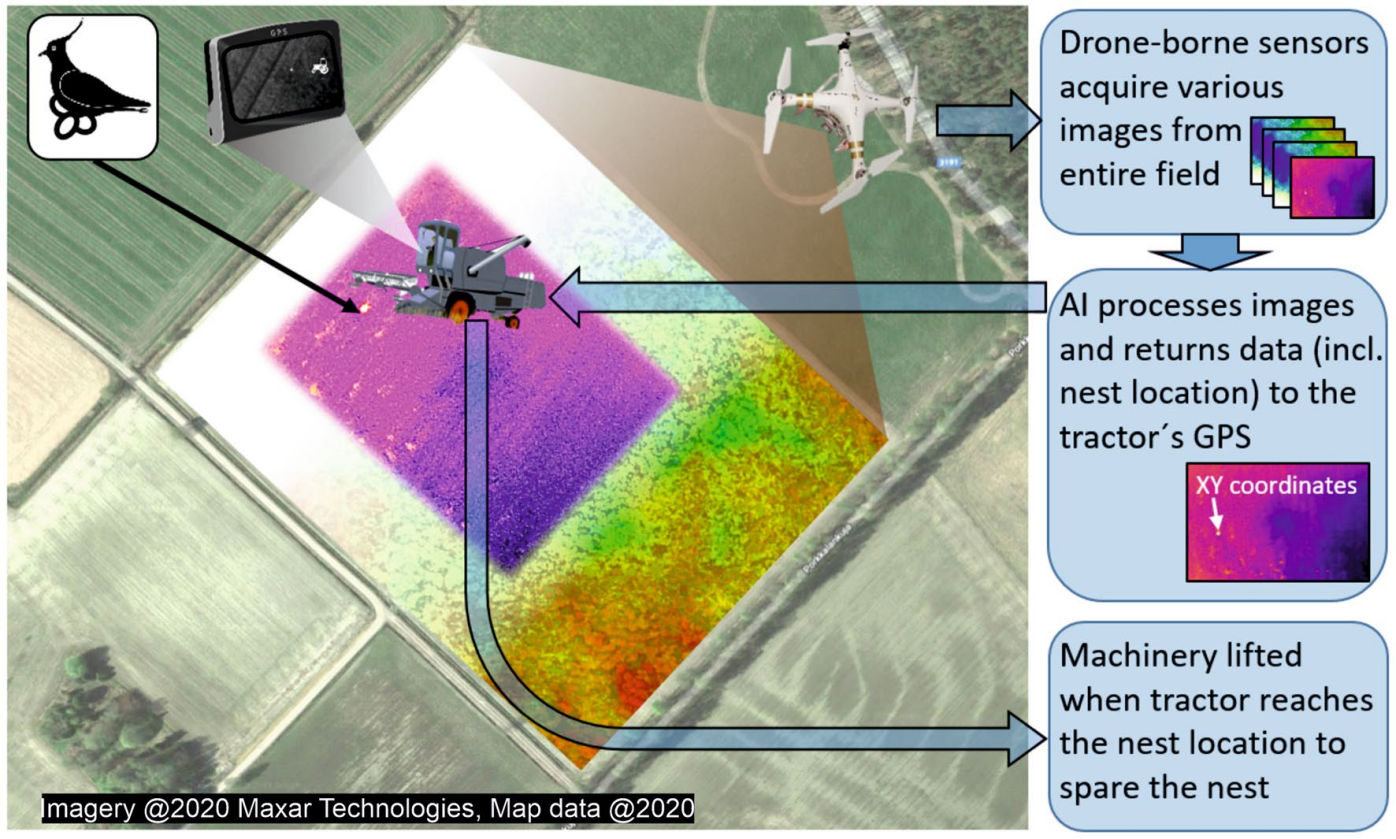
A schematic representation of the integration of a system for nest location within the practice of precision agriculture which makes extensive use of drones carrying sensors to map soil humidity, need for fertilizer and other chemical input and so on across fields. We envisage that thermal sensors can be added or complemented to the existing set in order to acquire thermal images that are processed by artificial intelligence (AI) specifically trained to automatically locate nests from thermal images. The geo-location of identified nests will then be transferred to the tractor that can be automatically programmed to avoid the nest through field operations, e.g. by changing direction or lifting the sower/harvester. The satellite image is taken from Google maps (www.google.it/maps, map data: Google). Figure created in Microsoft Office PowerPoint 2016 (www.microsoft.com).

We acknowledge that the system used here is semi-automated, with analysts’ time largely going into preparing learning material, and building and training the neural network algorithm. However, once training is done, the system becomes fully automated. Such a fully automated system will then allow real-time geo-location of ground nests, with potential to become an integral part of agricultural field monitoring performed, for example, within precision agriculture (Fig. 3). The integration of drone-borne sensors with information systems and advanced machinery of precision agriculture aims to enhance crop yield while minimizing environmental externalities, such as agro-chemical spillover, while reducing costs^13^. The shift towards this advanced way of food production is still in its infancy but progressing rapidly. The conservation community should be ready to embrace such a revolution by proposing solutions, such as the one presented here to automatically locate birds’ nests. However, for this to materialize, it is crucial that such automated wildlife and nest identification and location systems are widely tested and improved to reach high performance. This needs to be done with high urgency, because the pace of change in the means and technology for land use practices, such as agriculture, is very fast. Fundamentally, there is a need for the conservation community to switch from being technology users to become innovative leaders able to embrace technology to provide tools and solutions for conservation^15^.

Overall, we show that automated nest detection using drone-borne thermal imaging and deep learning is possible, and the performance is promising for wide applicability. Given the rise of drone use in conservation^7^, advances in drone and sensor technology and the resulting big data they can yield, it is fundamental that such systems are integrated with artificial intelligence approaches. Only by embracing the technological development, testing and proposing integrated tools and solutions, the conservation community will be able to keep pace with a rapidly changing world. This is challenging, but cases such as the one provided here, as well as several others^16^, pave the way to bridge the gap between technology and conservation^6,15^.

## Methods

### Ethics statement

The work was carried under the ethical Permit Number VARELY/711/2016 issued by the regional environmental centre of Finland. Every possible effort was made to minimise disturbance.

### Field system set-up

The study was performed in a farmland area in Southern Finland, near Lammi Biological Station (61° N, 25° E). We selected the lapwing as the study species, a common breeder in the study area. This ground nesting medium-sized farmland bird is listed as IUCN Near Threatened globally but Endangered within Europe^17^. The main threat to the species is agricultural intensification, and particularly the destruction of nests in arable land due to large scale mechanical operations, such as sowing^17^. In Finland, as in many other countries, a fraction of lapwing nests on arable land are located by volunteers, and their protection secured. However, locating these nests is highly time consuming and challenging, and most nests are left unprotected, and likely destroyed, every spring^18^.

**Figure 4:**
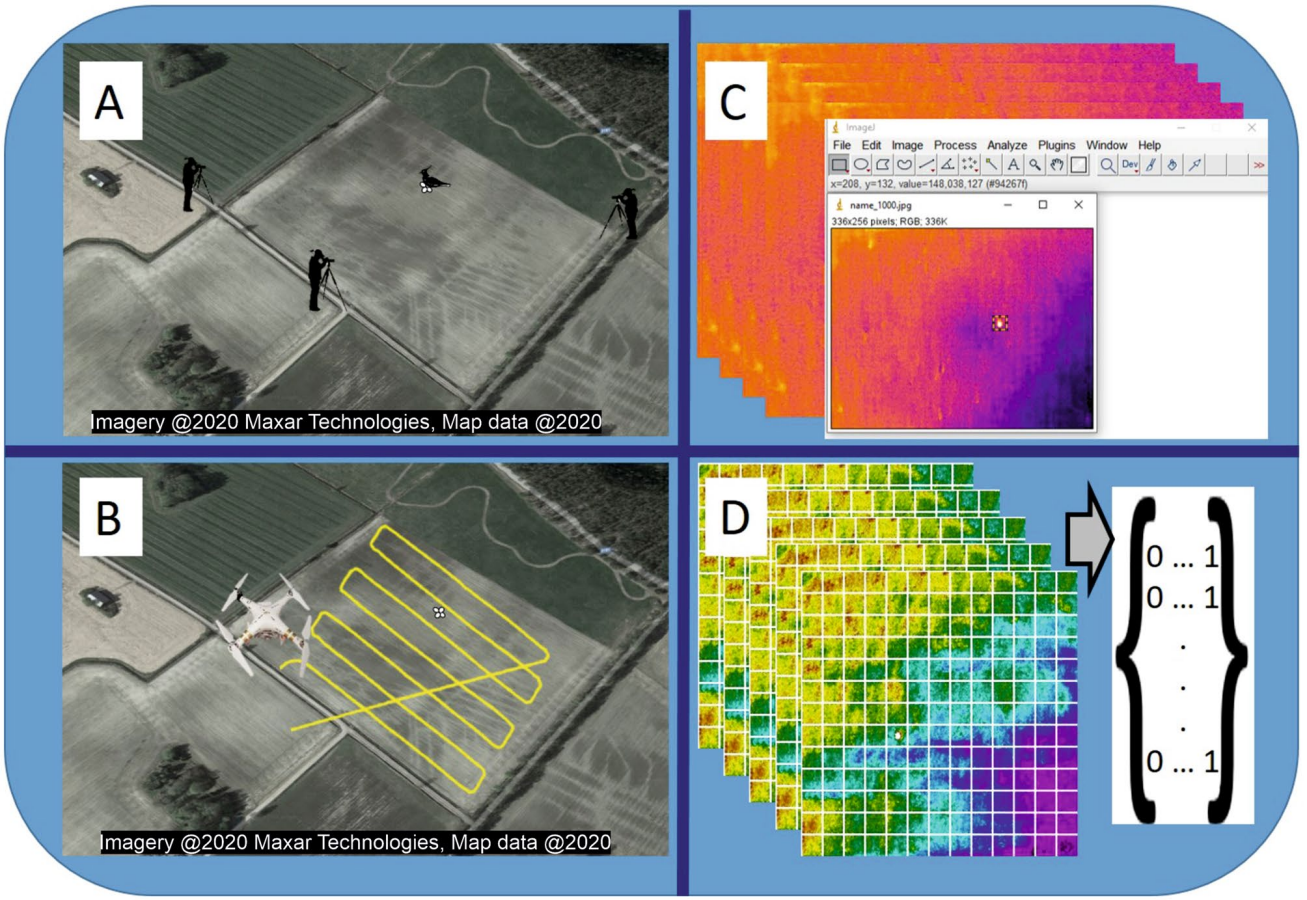
Schematic representation of the key steps of this study, from extensive nest search at selected fields (**A**), to flying the drone carrying the thermal sensor along a pre-programmed route over the field (**B**), preparing the thermal images by extracting the coordinates of the box drawn around the nest (**C**), and finally applying a neural network deep learning algorithm to classify images as having or not having a nest (**D**). The satellite image in (**A**) and (**B**) is taken from Google maps (www.google.it/maps, map data: Google). Figure created in Microsoft Office PowerPoint 2016 (www.microsoft.com).

We selected nine field parcels with a total of 22 lapwing nests in 2016, and three field parcels with nine nests in 2017. Study field parcels were intensively searched for nests by experienced observers during the week preceding the start of the recording work (see below), and individual nest locations were recorded with a hand-held GPS (model Garmin GPSmap 62 s, position accuracy ~ 3 m). Overall five of the field parcels were represented by ploughed fields (with irregular substrate owing to the upper soil being turned over) and seven by unploughed fields (direct sowing or winter cereals). Flight routes were constructed and saved in MapPilot 1.5.1 (Drones Made Easy, San Diego, USA) for areas around nests that covered approximately 0.5–1 ha (see Fig. 4). A drone cruised along the pre-programmed route, each flight lasting approximately 6–10 min (the time was constrained by the drone battery). Flights were done between 5-25.5.2016 and 16-18.5.2017 at altitudes of 15 or 25 m above ground level. Distance between transects was approximately 7 m for flights at 15 m of altitude and 12 m for flights at 25 m of altitude. We aimed to achieve a minimum of at least one flight over each field at each altitude (15 and 25 m) and each period of the day (07—11 a.m. and 14—20 p.m.). Overall, 54 flights were completed in 2016 and 19 in 2017. Thermal images were acquired by using a DJI Phantom 3 Advanced quadcopter with a FLIR Tau 2, 336 * 256 pixel, 9 mm lens thermal camera mounted on it. To ensure quality, thermal images were continuously saved during flights directly to a USB stick using a ThermalCapture unit (TeAx Technology GmbH, Wilnsdorf, Germany). Image acquisition occurred with the camera pointed in nadir position. Temperature, cloud cover, air humidity and wind speed data for each flight were accessed afterwards through Airdata.com service. In all circumstances, the incubating adult left the nest as we approached the edge of the field before the start of the drone flight.

### Deep learning algorithm for nest identification

To automatically classify images as having or not having a nest, we trained a deep learning model (more details in extended methods in Supplementary information) to detect nests while minimising the occurrence of false positives (hereafter false presences) and false negatives (hereafter false absences). In doing so, we choose a convolutional neural network (CNN) approach^19^ and YOLOv3 training program^20^, as done in a similar study^3^.

We selected all images with nests in them (positive images, n = 1969) from each flight, and a larger number of randomly selected images without a nest (negative images, n = 3,469). We manually labelled all positive images using software ImageJ 1.8.0^21^ with bounding boxes, which coordinates were then indexed by corresponding image names to be used by the YOLOv3. Next, we split the set of positive images into a training set and a test set by randomly selecting 70% of the flights (and all associated images) for training, and using the remaining 30% of flight images for testing. Selection was done at the flight rather than the image level to avoid that similar images from the same flight would be used in both training and testing, causing testing results to be positively biased.

Training was executed for 800 epochs, whereby an epoch is defined as the entire dataset passing forward and backward through the neural network (training program available at http://github.com/ultralytics/yolov3). After training was completed, a weights file was generated, which included the final value stored by each neuron, represented by floating point numbers in series. We then developed a program which read the configuration file of the YOLOv3 architecture and constructed the network according to the architecture specified in PyTorch implementation. Developing our own program was done to increase flexibility of expanding functionalities without interrupting unfamiliar code flow. The program read the trained weights file in series and stored the values to proper neurons, resulting in the construction of a deep learning model specifically for nest detection. This was then used to validate and test all the trained weight files to select the best model.

Through the testing, an output value was produced representing the confidence that an image included a nest. Values close to zero indicate that an image contained no nests with a high confidence, while values close to one that an image included a nest with a high confidence. In the cases where multiple spots appeared as possible nests, the overall confidence for that image was taken from the spot with the highest confidence values.

Performance of the neural network in discriminating images as having a nest or not was assessed by means of calculating four standard evaluation metrics for these types of systems: Precision, recall (propensity to avoid false positives and false negatives, respectively), accuracy (overall discrimination accuracy) and F1 score (the harmonic mean of precision and recall; more details in extended methods in Supplementary information). Performance was evaluated separately for the training and for the testing parts of the system.

### Statistical analyses

We quantified the effect of environmental factors on the performance of the deep learning algorithm. We ran two separate analyses, one focused on factors affecting occurrence of false presences (i.e. the algorithm erroneously identifies an image without a nest as having a nest), and one on factors affecting occurrence of false absences (algorithm erroneously identifies an image with a nest as not having a nest). As the response of the former model we used the confidence as given by the algorithm for all the test images known to have no nest in them (values close to one indicate high probability of false presence). This model used data from images without a nest from all available flights (n = 3,469 images from 73 flights, as these were not used for training). The response for the false absence model was the confidence of the test images (n = 497 images with a nest from the 30% flights, n = 14 flights, not used for training) to having no nest in them (values close to one indicate high probability of false absence). Because the response variable is a proportion, we used beta regression with logit link. In each model we also included flight identity as a random factor to account for pseudo-replication stemming from multiple images per flight.

Models were run using the glmmTMB package^22^ in R version 3.6.1^23^. Covariates considered are: air temperature (hereafter temperature), cloud cover, wind speed, air humidity (hereafter humidity), drone height (either 15 or 25 m above ground level) and substrate type (two classes: ploughed or un-ploughed). These variables are deemed potentially relevant in affecting the nest visibility in thermal images, and consequent performance of the deep learning algorithm. Air temperature, humidity, cloud cover and wind speed may reduce the thermal contrast between a nest (warm) and the remaining landscape, and create heterogeneous thermal landscape that hampers nest detection^24^. Moreover, ploughing alters the physical structure of the substrate, making it rougher and more heterogeneous, with many physical objects that may resemble a nest shape, thereby affecting nest detection accuracy. Drone height may affect detection accuracy due to the decreasing size and sharpness of a nest at higher altitudes.

Prior to analyses, we checked for outliers and ran variance inflation (VIF) analyses to assess the collinearity level between covariates. For the false presence model, humidity had a high VIF value of 3.9 being strongly correlated to temperature (R = − 0.7) and was excluded from following analyses. For the false absence model, humidity and wind speed had high VIF (28.8 and 2.3, both correlated with temperature, R = − 0.9 and 0.7, respectively), and were excluded from analyses. The remaining covariates had a VIF < 2, indicating low collinearity^25^. We then constructed a full model for each of the two response variables and performed model selection and multimodel averaging based on the set of best supported (with 95% confidence) models as given from Akaike weights^26^ using the MuMin package^27^.

## Data availability

The datasets collected and analysed in the current study including thermal imagery are available from the corresponding author upon request.

## Supplementary information

Supplementary file1 (PDF 641 kb)
