# Supplementary material for: Integrating drone-borne thermal imaging with artificial intelligence to locate bird nests on agricultural land
Source: Sci Rep. 2020 Jul 14;10:10993. doi: 10.1038/s41598-020-67898-3 (PMC7360548; doi:10.1038/s41598-020-67898-3)
Supplement: Supplementary file 1 — Supplementary file1 (PDF 641 kb) [file 41598_2020_67898_MOESM1_ESM.pdf]

**Integrating drone-borne thermal imaging with artificial intelligence to locate bird nests on agricultural land**

*Andrea Santangeli[1,2] *, Yuxuan Chen[3,4], Edward Kluen[5,6], Raviteja Chirumamilla[3,7], Juha Tiainen[8], John Loehr[3]*

*Correspondence to: andrea.santangeli@helsinki.fi; tel: +358 504484443; fax: +358 2941 57694

# SUPPLEMENTARY INFORMATION

## Extended methods, figures and tables

**Deep learning process details:**

YOLOv3 (Redmon and Farhadi, 2018) is a fully convolutional network (FCN), using only convolutional layers with shortcut connections and bilinear upsampling layers. Classical convolutional networks include another FCN at the end of the network to receive features gained from previous convolutional layers and make final predictions, which damps speed performance, but with the rationale that final feature maps are small enough for an FCN to process.

To avoid the slower part, YOLOv3 uses 1 × 1 convolutional layers at the end to output predictions. This means the final output itself is just another feature map that can represent object attributes. Another characteristic of YOLOv3 is that no pooling is used in YOLOv3, but convolutional layers of stride (downsampling factor) 2 are used to downsample feature maps. Convolutional layers have an advantage over pooling in that they prevent the loss of low-level features that occurs when pooling takes only the maximum value in its receptive field, whereas convolution combines every value in the receptive field. This makes it suitable for our research to detect the presence of nests, which are small objects.

A feature map consists of multiple cells with depths. In YOLOv3, each cell can predict 3 candidate bounding boxes. For example, if the size of input images is 128 × 128, and network stride is 32, the output will be 4 × 4, meaning the output feature map has 16 cells and 48 candidate boxes.

The depth represents attributes of each bounding box, which includes center x- and y-coordinates, length, width, objectness score, which measures how likely an object is contained in the bounding box, and confidence scores for C classes respectively. Therefore, each cell has depth (3 × (5+C)).

We kept input size constant for processing convenience and GPU batch processing, so we resize all input images to 416 × 416 using bicubic interpolation. With network stride being 32, the output is then 13 × 13, so it is divided into a grid of 13 × 13, corresponding to the 13 × 13 output feature map (Figure 1).

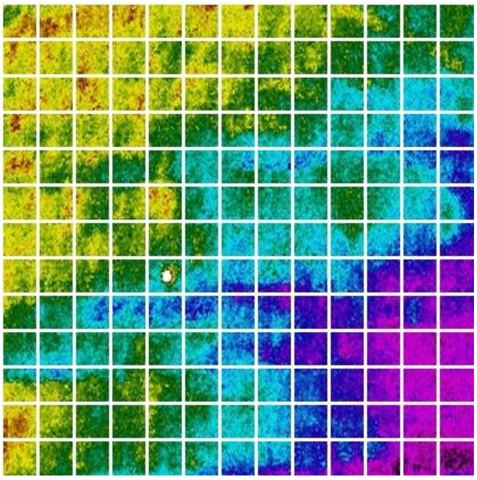

**Figure S1**. Example of a thermal image with a 13 × 13 grid used for detecting nests. The nest is visible in column 5 (numbered starting from left) and row 8 (numbered starting from the top).

A cell will predict an object if the object center lies in the receptive field of that cell, so the cell at 5th column and 8th row is assigned responsible for detecting the nest. However, the same cell will generate 3 detection proposals, but only the one with highest IoU (Intersection over Union, a metric to evaluate accuracy commonly used for FCN) with the ground truth box is chosen as the final prediction. In case of multiple detections, it first thresholds based on objectness score and then uses non-maximum suppression (NMS) based on class confidence scores to ignore all irrelevant detections.

References

Redmon, J., and A. Farhadi. 2018. YOLO v3: An Incremental Improvement. arXiv preprint arXiv:.08242.

**Algorithm performance evaluation and details:**

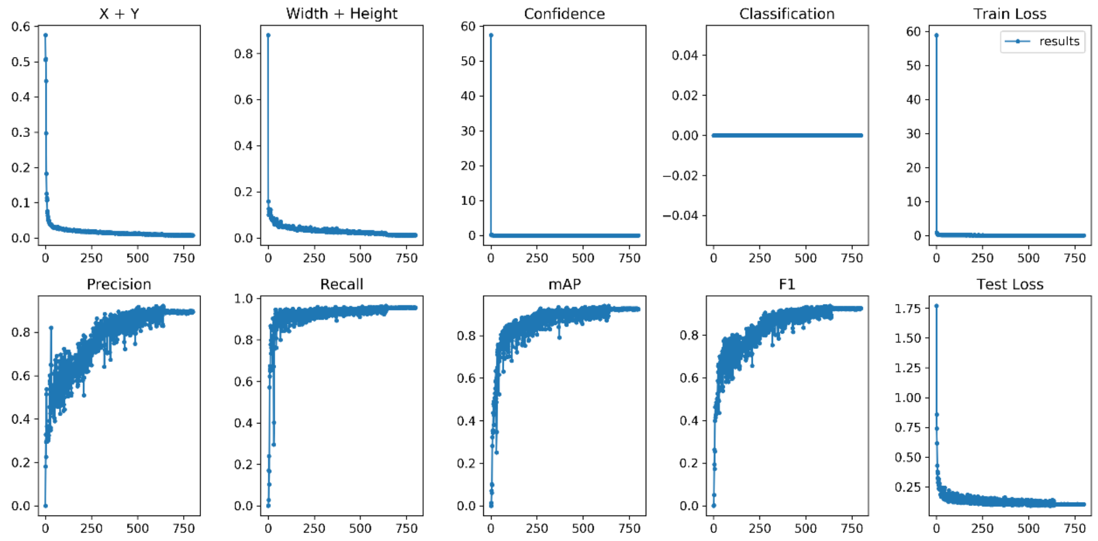


**Figure S2.** Overall performance evaluation metrics of the learning for the neural network system (see text below for details on each panel and the metrics they represent).

Top 5 panels of Figure S2 show the variation of training loss with respect to training epochs, where loss is a term used in machine learning to indicate error, e.g. mean square error (MSE), between the network output and ground truth. Since the bounding boxes predicted by the network are represented by their center coordinates and sizes, the loss of bounding box prediction is represented by X+Y and Width+Height (shown in the two leftmost top panels). Each bounding box will then assign a class to the object contained inside the bounding box and how likely the object belongs to that class (shown by the top panels titled Confidence and Classification of Figure S2). Note that the classification panel stays at zero error throughout the process because the only possible positive output is "nest", but no other options are available. The rightmost top panel in Figure S2 represents training loss through the overall MSE by taking all the network outputs.

Bottom panels in Figure S2 represent various performance metrics evaluated after each epoch trained using the same training data. By comparing the predictions to the ground truth, each detection is concluded as true positive, false positive, true negative or false negative.

Precision is the fraction of relevant instances among retrieved instanced, defined as

$$Precision = \frac{True\ Positives}{True\ Positives + False\ Positives}$$

Recall is then the fraction of relevant instances that are correctly retrieved, defined as

$$Recall = \frac{True\ Positives}{True\ Positives + False\ Negatives}$$

F1 is the harmonic mean between precision and recall, defined as

$$F1 = \left(\frac{Precision^{-1} + Recall^{-1}}{2}\right)^{-1} = 2 * \frac{Precision * Recall}{Precision + Recall}$$

This metric is needed because there exists a false negative-false positive tradeoff which results in the precision-recall tradeoff. By taking their harmonic mean, F1 can give a performance metric that is less subject to this tradeoff.

mAP is best explained by giving an example. Let the following table show the prediction of an image, which is predicted to contain 4 positives, out of which only 1 is the true positive, we then have the table below to indicate if each detection is relevant (true positive) or not. Precision at each rank is then obtained by the definition above, and average precision can be then the calculated by the average of precisions at all ranks, and mAP is finally obtained by taking average of all images' average precisions.

| Nest detected (rank k) | Relevant (true positive or not) | Precision at rank k |
|---|---|---|
| 1 | N | 0/1=0 |
| 2 | Y | 1/2=0.5 |
| 3 | N | 1/3=0.33 |
| 4 | N | 1/4=0.25 |
| | Average Precision: | (0+0.5+0.33+0.25)/4=0.27 |

The final test loss panel (rightmost bottom panel in Figure S2) shows the overall retrieval error, i.e. by classifying results into true positive, false positive, true negative, false negative, using MSE with ground truth. Note that test loss is different from the training loss panel above, as training loss is used for backpropagation to update the network in the next epoch, while test loss purely serves as a performance metric. Nevertheless, as training proceeds, all the metrics are expected to grow and converge to a high value, while loss is expected to decrease and converge to a low value.

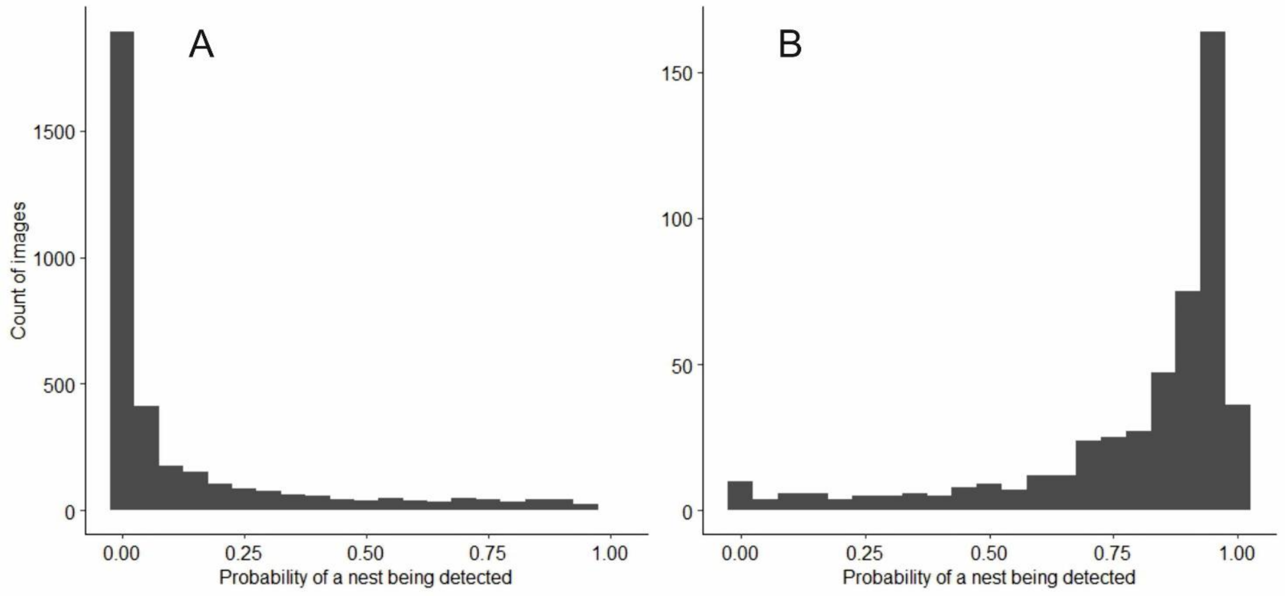


**Figure S3.** The frequency distribution of the probabilty of an image to have a nest in it as assigned by the deep learning algorithm for (A) images that do not include a nest (n = 3469), and (B) images that do include a nest (497). These images were never seen by the algorithm for learning, and were used in the statistical models as presented in Table 1 of the main manuscript.

.**Table S1.** The 95% confidence set of best supported models assessing the effect of a set of environmental covariates on the occurrence of false presences (A) and false absences (B). Results from these models were then averaged through the multi-model averaging step to derive the statistics presented in Table 1 of the main manuscript. (Null) indicates the null model, whereby only the intercept was included.

| **A) False presence** | **df** | **AIC** | **ΔAIC** | **AIC weight** |
|---|---|---|---|---|
| Cloud cover + Substrate + Temperature | 6 | -12961.98 | 0.00 | 0.33 |
| Cloud cover + Substrate + Temperature + Wind speed | 7 | -12960.93 | 1.05 | 0.19 |
| Cloud cover + Drone height + Substrate + Temperature | 7 | -12960.38 | 1.61 | 0.15 |
| Cloud cover + Drone height + Substrate + Temperature + Wind speed | 8 | -12959.35 | 2.64 | 0.09 |
| Cloud cover + Temperature | 5 | -12958.92 | 3.07 | 0.07 |
| Cloud cover + Substrate + Wind speed | 6 | -12957.91 | 4.07 | 0.04 |
| Cloud cover + Temperature + Wind speed | 6 | -12957.73 | 4.25 | 0.04 |
| Cloud cover + Drone height + Temperature | 6 | -12957.24 | 4.75 | 0.03 |
| Cloud cover + Wind speed | 5 | -12956.84 | 5.14 | 0.03 |
| Cloud cover + Drone height + Substrate + Wind speed | 7 | -12956.07 | 5.91 | 0.02 |
| Cloud cover + Drone height + Temperature + Wind speed | 7 | -12956.07 | 5.92 | 0.02 |
| **B) False absence** | | | | |
| Drone height + Temperature | 5 | -675.43 | 0.00 | 0.16 |
| Drone height | 4 | -675.20 | 0.23 | 0.15 |
| Drone height + Substrate + Temperature | 6 | -675.06 | 0.37 | 0.14 |
| Drone height + Substrate | 5 | -674.27 | 1.15 | 0.09 |
| Cloud cover + Drone height + Temperature | 6 | -673.78 | 1.64 | 0.07 |
| (Null) | 3 | -673.78 | 1.65 | 0.07 |
| Temperature | 4 | -673.31 | 2.12 | 0.06 |
| Cloud cover + Drone height | 5 | -673.30 | 2.13 | 0.06 |
| Cloud cover + Drone height + Substrate + Temperature | 7 | -673.23 | 2.20 | 0.05 |
| Substrate | 4 | -672.71 | 2.72 | 0.04 |
| Substrate + Temperature | 5 | -672.62 | 2.81 | 0.04 |
| Cloud cover + Drone height + Substrate | 6 | -672.29 | 3.13 | 0.03 |
| Cloud cover | 4 | -671.86 | 3.57 | 0.03 |
